# Modelling Temporal Stability of EPI Time Series Using Magnitude Images Acquired with Multi-Channel Receiver Coils

**DOI:** 10.1371/journal.pone.0052075

**Published:** 2012-12-20

**Authors:** Chloe Hutton, Evelyne Balteau, Antoine Lutti, Oliver Josephs, Nikolaus Weiskopf

**Affiliations:** 1 Wellcome Trust Centre for Neuroimaging, UCL Institute of Neurology, University College London, London, United Kingdom; 2 Cyclotron Research Centre, University of Liege, Liege, Belgium; University of California San Francisco, United States of America

## Abstract

In 2001, Krueger and Glover introduced a model describing the temporal SNR (tSNR) of an EPI time series as a function of image SNR (SNR_0_). This model has been used to study physiological noise in fMRI, to optimize fMRI acquisition parameters, and to estimate maximum attainable tSNR for a given set of MR image acquisition and processing parameters. In its current form, this noise model requires the accurate estimation of image SNR. For multi-channel receiver coils, this is not straightforward because it requires export and reconstruction of large amounts of k-space raw data and detailed, custom-made image reconstruction methods. Here we present a simple extension to the model that allows characterization of the temporal noise properties of EPI time series acquired with multi-channel receiver coils, and reconstructed with standard root-sum-of-squares combination, without the need for raw data or custom-made image reconstruction. The proposed extended model includes an additional parameter κ which reflects the impact of noise correlations between receiver channels on the data and scales an apparent image SNR (SNR′_0_) measured directly from root-sum-of-squares reconstructed magnitude images so that κ = SNR′_0_/SNR_0_ (under the condition of SNR_0_>50 and number of channels ≤32). Using Monte Carlo simulations we show that the extended model parameters can be estimated with high accuracy. The estimation of the parameter κ was validated using an independent measure of the actual SNR_0_ for non-accelerated phantom data acquired at 3T with a 32-channel receiver coil. We also demonstrate that compared to the original model the extended model results in an improved fit to human task-free non-accelerated fMRI data acquired at 7T with a 24-channel receiver coil. In particular, the extended model improves the prediction of low to medium tSNR values and so can play an important role in the optimization of high-resolution fMRI experiments at lower SNR levels.

## Introduction

The optimization of EPI acquisition parameters is important for maximizing the sensitivity to relatively small BOLD signal changes in fMRI studies. Factors such as spatial resolution, echo time (TE), flip angle, and parallel imaging strategies can be optimized for a given magnetic field strength and receiver coil arrangement. In particular, these factors can influence the contribution of physiological noise, for example from cardiac and respiratory functions as well as motion, to the temporal noise, thereby impacting on BOLD sensitivity. The noise model introduced by Krueger and Glover [1] describes the temporal signal to noise ratio (tSNR) of an EPI time series as a function of image SNR and has been used to demonstrate that the ratio of physiological noise to thermal noise increases with image SNR [1]–[3]. The model allows the time series noise to be separated into thermal and physiological components and can be used to estimate the maximum attainable tSNR for a given set of MR acquisition parameters [2] and processing strategies [4].

In its current form, the application of this noise model requires an accurate estimate of the actual image SNR (SNR_0_). The estimation procedure is particularly elaborate for images acquired with multi-channel receiver coils, involving detailed steps to correctly scale the image SNR [3], [5]. Further steps are necessary for images reconstructed using partial parallel imaging methods [3], [5]. Some of the main factors that must be taken into account are noise correlation between receiver channels, change of statistical distributions in the case of magnitude images, geometry factors in under-sampled parallel imaging and temporal autocorrelation in the k-space raw data [5]. Although a principled way to reconstruct and output images in SNR units is proposed in [5], this is not part of the standard image reconstruction available on clinical MRI scanners. Thus, SNR_0_ can only be correctly estimated by custom-made image reconstruction based on k-space raw data. This approach is inefficient and in many cases even impossible, since it requires export, storage and computation of large amounts of raw data (e.g. ∼70 GB for ∼10 minutes of high-resolution fMRI acquired using a 32-channel receiver coil).

A common approach to combining information from multiple receiver channels is to calculate the root-sum-of-squares (RSS) across channels, after reconstructing the image separately for each channel. This method is regularly used for non-accelerated image acquisition [6] and parallel imaging employing GRAPPA [7]. It has been demonstrated [8] that the image SNR can be estimated from the background noise and image signal in RSS combined non-accelerated images using a simple correction for the change in statistical distributions due to rectification of multiple channel data, i.e. the noncentral chi distribution. However, this approach is only valid if no noise correlations between channels are present, which is generally not the case for RSS composite images acquired with state-of-the-art multi-channel RF head coils [3], [9]. In fact, as pointed out in [5], correctly estimating SNR has caused significant confusion and discussion in the community. For example, the SNR correction methods proposed in [10]–[12] account only for the RSS combination and magnitude operation. This correction does not take into account noise correlations in the data from the different receiver channels as explained in [8]. Typical levels of noise correlation (i.e. ∼0.2 to 0.3 [9]) cause a spatial variation of the noise in the composite images [6], [13], [14] and lead to errors in SNR measurement when noise correlations are not taken into account. To overcome this problem, the methods described in [5] and [3] use the noise covariance matrix to account for correlations between different channels when estimating the SNR and are in line with the formulation of [6].

In this study, we extend the model proposed in [1] to allow the tSNR of an EPI time course acquired without acceleration using multi-channel receiver coils to be characterized, without the necessity for complex custom-made image reconstruction of absolute image SNR estimates. Instead, the straightforward approach described in [8] is used for estimating an apparent image SNR′_0_ and the impact of noise covariance between receiver coils is accounted for by introducing a constant scaling factor κ = SNR′_0_/SNR_0_, which modulates the actual image SNR_0_. To our knowledge, no other published methods can estimate the parameters of the model proposed in [1] from RSS combined magnitude data acquired using multi-channel receiver coils. We derive our proposed extended model from basic physical principles and use Monte Carlo simulations to optimize and demonstrate the accuracy and precision of the resulting fit. We validate the extended model fit using phantom data acquired at 3T and an independent estimate of the actual SNR_0_. Finally we demonstrate the improved fit of the extended model in human task-free fMRI data acquired at 7T.

### Theory

Following the theory outlined in [1] the total variance or noise in an MR image time series σ^2^ can be described by a combination of independent raw noise σ_0_^2^ and physiological noise σ_p_^2^:
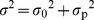
(1)


The noise variance σ_0_^2^ is assumed to comprise thermal noise from the subject and scanner electronics. The physiological noise σ_p_^2^ is assumed to arise from cardiac and respiratory functions, which lead to oscillatory signal fluctuations in the vascular system as well as small pulsatile movements of the brain and modulation of the magnetic field. It has been shown that σ_0_ increases with field strength [15] but is independent of the MR signal strength, whereas physiological noise has been shown to be signal-dependent [1]. Note that in [1] the physiological noise is treated as two separate components. One describes T2* related fluctuations, which are TE-dependent and also lead to the BOLD effect and the other arises from image-to-image fluctuations such as pulsatile effects and scanner imperfections with no TE-dependencies. For the purpose of the theory developed here, we do not make a distinction between these components of physiological noise and only consider the case of physiological noise represented by σ^2^_p_. Therefore, following on from [1] and the formulation used in [2], the temporal SNR (tSNR) can be defined as
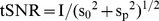
(2)where I is the mean image signal intensity. If the actual image SNR is defined as SNR_0_ = I/σ_0_ and the physiological noise is defined as a signal dependent function σ_p_ = λI, then the relationship between tSNR and SNR_0_ is given by [2]:



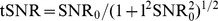
(3)The parameter 1/λ provides a measure of the maximum attainable tSNR for a given set of acquisition parameters. It can be estimated by measuring tSNR for different values of the actual image SNR_0_ (e.g. by varying flip angle, voxel size or echo time). From equation 3 it can be seen that, in the absence of physiological noise, i.e. when λ = 0, tSNR = SNR_0_. Equation 3 requires an accurate, unbiased estimate of SNR_0_ as discussed above.

In the following we extend the model so that instead of the actual SNR_0_, the apparent SNR′_0_ measured from RSS combined magnitude images using the approach proposed by [8] can be used. For this method the SNR′_0_ is the ratio of the signal intensity in the measured object to the noise level in the background and equals the actual SNR_0_ in the absence of noise correlations.

For a receiver system with *n* coils a measure of background noise σ′_0_ in RSS combined images can be estimated using (equation 7 in [8]):
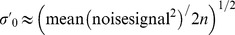
(4)


**Figure 1:**
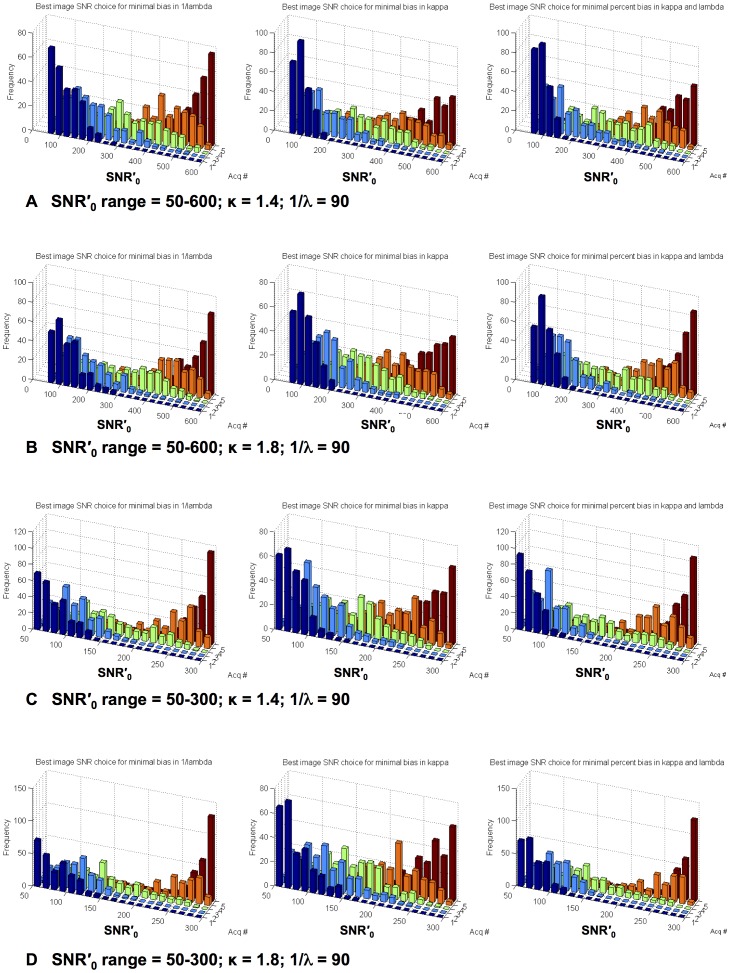
Best sets of 5 SNR′_0_ values for which the bias in κ and 1/λ is minimized, as determined from Monte Carlo simulations. The results for different parameters λ = 1/90, κ = 1.4/1.8, SNR′_0_ ranges = 50–600/50–300 are presented as histograms of the top 5% best sets of SNR′_0_ in (A–D). Acq # orders the 5 acquisitions according to their SNR′_0_. It can be seen that these best sets sample low and high SNR’ values more densely. For the small SNR′_0_ range (C, D), high SNR′_0_ values are more strongly represented, in order to better estimate 1/λ.

It is important to note that although this noise estimate includes the corrections for the RSS combination and magnitude operation (i.e. the noncentral chi distribution), it does not take into account noise correlations and is therefore only accurate in their absence. Equation 4 is therefore correct for data acquired using a single-coil (which of course can not suffer from noise correlations) but not for multi-channel receiver coil data with noise correlations between receiver channels. We therefore use equation 4 to define the apparent image SNR as, SNR′_0_ ≈ I*_n_*/σ′_0_, where I*_n_* is the mean signal in the RSS combined images. For low SNR′_0_ further corrections must be applied to account for changes in the noise statistics. We will neglect this correction in the following since they are less than 2% for image SNR exceeding 50 and number of receiver channels not exceeding 32 [3], (i.e. the currently maximum number of channels in commercially available coils). These additional corrections for the noise statistics are only required at even lower SNR values for data acquired with less than 32 channels (Figure 1b, [3]).

In general SNR′_0_ only equals the actual image SNR_0_ for data acquired with a single-channel receiver coil. When *n* ≥2 receiver coils are used to acquire array coil images and if noise correlations exist between the different channels of the receiver system, the actual variance σ*_In_^2^* in a voxel with composite intensity *I_n_* will deviate from the background noise variance estimate σ′_0_^2^ [13], [14]. We therefore define a constant scaling factor κ to account for correlated noise, which relates SNR_0_ and SNR′_0_ in a voxel by:
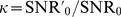
(5)


Replacing SNR_0_ by SNR′_0_ in equation 3 yields our proposed extended model:
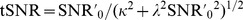
(6)where κ and λ can be estimated from equation 6 by measuring tSNR for different values of SNR′_0_ (i.e. in a similar way as for equation 3). Note that because correlated noise is spatially varying, κ will also vary spatially over an image. Furthermore, we assume that κ will be dependent to some extent on the loading of the coil and so will vary for phantoms and human subjects. In this study we will therefore demonstrate the validity of equations 5 and 6 in phantom and human data.

## Methods

### Accuracy and Precision of Extended Model Fit Estimated from Monte Carlo Simulations

The estimation of λ and κ from the measured SNR′_0_ and tSNR data poses a non-linear problem, which was solved by a multidimensional unconstrained nonlinear minimization (Nelder-Mead) method in Matlab (2009a, The MathWorks, Natick, MA). To address potential problems with model estimability, Monte Carlo simulations were performed to study the accuracy and precision of the estimation of κ and λ, and to determine how a set of five SNR′_0_ values should be best distributed to obtain fit parameters with highest accuracy.

For the simulation, synthetic tSNR values were generated using the extended model (equation 6) with a realistic set of parameters: λ = 1/90, κ = 1.4 or 1.8 and five evenly distributed SNR′_0_ values in the range of 50 to 600 or 50 to 300. These SNR′_0_ values are typical for 7T and 3T data respectively; SNR′_0_ values <50 were excluded, since they would require further correction for changes in the noise distribution [8], if 32 or more channels were used. Gaussian distributed noise with zero mean and a standard deviation of 5 (typical for group studies [2], [4]) was added to the tSNR values to simulate measurement noise. The model was then fitted to this set of synthetic data, yielding model parameters λ and κ. This process was repeated 500 times to get a reliable estimate of the mean and standard deviation (SD) of the fitted model parameters λ and κ.

To determine how a set of five SNR′_0_ values should be best distributed to obtain unbiased and low noise estimates of λ and κ, the above simulation was repeated 5000 times, each with a different set of five arbitrary SNR′_0_ values. Each repetition resulted in an estimate of λ and κ calculated using five unique SNR′_0_ values, which were selected randomly from the given range based on a uniform probability distribution. From the 5000 sets of five SNR′_0_ values, 250 were selected (i.e. five percent), for which the estimate of λ and κ had the lowest percent bias. For these 250 estimates of λ and κ values, the accuracy was calculated from their mean absolute bias and the precision from the SD. For visual exploration, histograms of the best 250 sets were generated to show the distribution of each of the five SNR′_0_ values in increasing order (Figure 1). The precision of the estimates based on the best sets was estimated from the mean SD over the sets. For comparison, the sets with the highest precision out of the 5000 sets and their SD were also determined, providing an approximate upper limit of the highest precision achievable (regardless of accuracy).

An additional simulation explored the model behaviour for phantom data. Temporal SNR measurements on a gel phantom are usually not significantly affected by signal dependent noise if standard acquisition parameters are used due to the high temporal stability of modern MRI scanners. Thus, the tSNR is small compared to 1/λ for the phantom and the tSNR is not saturated. To determine whether κ can be estimated robustly under these conditions, the same Monte Carlo simulations described previously were repeated with the following parameters: λ = 1/1800; SNR_0_ = (SNR′_0_/κ) = 60 or 120 or 180 for κ = 1.0 to 2.0 (increased in steps of 0.1). The parameters were typical for phantom measurements as determined from independently estimated SNR_0_ maps (see below) and quality assurance experiments [16], [17].

The third type of Monte Carlo simulation addressed whether λ and κ can be estimated robustly without relevant correlation between the parameters. If the non-linear minimization problem is ill posed, the estimated parameters may be spuriously correlated, skewing the results. The correlation between parameters were estimated for a measurement approximating the in-vivo dataset acquired at 7T with SNR_0_ = (SNR′_0_/κ) = 80, 180, 270, 350, 450. In one simulation κ was varied from 1 to 2 (in steps of 0.0001) and the effect on the estimation of λ was studied. In the second simulation 1/λ was varied from 80 to 140 (in steps of 0.005) and the effect on the estimation of κ was studied. As in the previous simulations Gaussian distributed noise with zero mean and a standard deviation of 5 was added to the tSNR values for each simulated 1/λ or κ set. Robust regression analysis (iteratively reweighted least squares with the bisquare weighting function as implemented in Matlab) was used to assess whether there was a relevant correlation between the parameters.

### Validation of Extended Model Fit using Phantom Data and Independent Estimate of Actual Image SNR

We used EPI time series acquired from a phantom to confirm that the scaling factor κ estimated from the extended model (equation 6) equals the ratio SNR′_0_/SNR_0_ (i.e. to validate equation 6) where SNR′_0_ is estimated from RSS combined images and SNR_0_ is an independent estimate of actual image SNR, according to [3], [5]. An agar gel phantom (construction based on the Stanford Agar Phantom Recipe [16]) was scanned using a 3T whole-body MRI scanner (Magnetom TIM Trio, Siemens Healthcare, Erlangen, Germany) operated with a 32-channel RF head receive and RF body transmit coil. Three EPI runs were acquired with different RF excitation flip angles to manipulate the image SNR. Each run comprised of 205 volumes and the flip angles were 17°, 37° and 90°. A thermal noise measurement of 20 EPI volumes with no RF excitation (i.e. 0° flip angle) was also acquired. These flip angles resulted in images with equally spaced image SNR levels from 0° up to a maximum at 90°, which is approximately the Ernst angle for gray matter at 7 T for the used TR [20].

The EPI data were collected with the following parameters [18]: matrix = 64×74, in-plane resolution = 3 mm×3 mm, 49 sequentially acquired slices, slice thickness = 2 mm, interslice gap = 1 mm, TE = 30 ms, volume TR = 3.43 s, echo-spacing = 500 µs, BW = 2298 Hz/Px. At the beginning of the experiment the manufacturer’s automatic adjustment procedure was performed to correct for first and second order distortions in the static magnetic field. The k-space raw data from all time series were reconstructed using an algebraic trajectory-based reconstruction approach designed to minimize ghosting [19] followed by RSS calculation to combine the images from the 32 receiver channels. The resulting magnitude images were then processed using routines implemented in Matlab.

A measure of noise (σ′_0_) was estimated from a background ROI from the RSS images acquired without RF excitation (i.e. with 0° flip angle) using equation 4. According to [8], this particular noise measure is not affected by noise correlations. However, since in general this assumption can be violated, we use this noise measure to calculate apparent image SNR using SNR′_0_ = I*_32_*/σ′_0_ where I*_32_* is the mean signal at each voxel in the RSS combined images. The tSNR was calculated using tSNR = I*_32_*/σ*^t^_I32_* where σ*^t^_I32_* is the temporal standard deviation at each voxel. Voxelwise maps of image SNR and tSNR were calculated for each flip angle time series after discarding the first 5 volumes and correcting for low frequency temporal drifts by fitting and removing a linear and quadratic function of image number. The extended model defined in equation 6 was fitted to the tSNR and SNR′_0_ values at each voxel in the central slice of the phantom resulting in maps of λ and κ values.

The independent estimate of SNR_0_ was based on an established method to estimate actual image SNR for RSS combined images using the individual channels of k-space raw data [3], [5], [6]. The SNR_0_ at each voxel in the RSS combined image is given by.
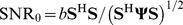
(8)where **S** is the vector of complex image values across all coils for a given voxel, **S** ^H^ is the Hermitian transpose of **S,** **Ψ** is the noise covariance matrix and *b* is a correction factor to account for the algebraic reconstruction method used to transform the k-space raw data to images and autocorrelation in k-space data as well as other effects, e.g. see [3], [5]. The complex valued trajectory-based reconstructed data from each channel in the first non-discarded image of each flip angle EPI time series formed the vector **S**. **Ψ** was calculated from the covariance between all pairs of channels of k-space raw data acquired as the thermal noise measurement (i.e. acquired with no RF excitation). The correction factor *b* was estimated from the square root of the ratio between the integrated power in the k-space raw noise data and the trajectory-based reconstructed noise data. Note that this estimate for SNR_0_ takes into account noise correlations between receiver channels. Resulting maps of SNR_0_ were compared with maps of tSNR, SNR′_0_ and κ values estimated from the RSS combined EPI time series. The extended model was also fitted to the tSNR and SNR_0_ values to estimate λ and κ.

### Fit of Extended Model to Human task-free fMRI Data

A 7T whole-body MRI scanner (Siemens Healthcare) using a 24-channel receive head coil with dedicated CP coil for RF transmission (Nova Medical, Inc., Wilmington, MA) was used to acquire EPI time series from 5 subjects. Written informed consent was obtained from each participant for the study approved by the ethics committee of the University of Magdeburg. The data were acquired as part of a comprehensive study to investigate the impact of physiological noise correction at 7T (further details of the full study are reported in [4]).

For each subject, 5 EPI runs were acquired while subjects were presented with a blank screen and instructed to rest with their eyes open. Each run comprised 150 volumes and was acquired with one of the following flip angles: 8°, 16°, 26°, 38° and 70°, selected in a randomized order. These flip angles were selected to produce images with an equally spaced range of signal levels from 0° up to a maximum at 70°, the Ernst angle for grey matter at 7 T at a TR = 2 s, (assuming T1 = 1.9 s [20]). At higher field strengths it is important to account for large B1 inhomogeneities [21] when setting the RF excitation flip angle to maximize the image SNR in a particular region of interest. Here the scanner adjustment procedures resulted in an actual RF excitation flip angle, which was reasonably close (within ∼10%) of the nominal flip angle in the studied region of interest, i.e., visual cortex. A thermal (raw) noise measurement of 20 EPI volumes with no RF excitation (i.e. 0° flip angle) was also acquired.

At the beginning of the experiment the manufacturer’s automatic adjustment procedure, optimized for 7T, was performed to correct for first and second order distortions in the static magnetic field and to set the RF transmitter voltage. The EPI data were collected with the following parameters [18]: matrix = 64×64, in-plane resolution = 3 mm×3 mm, 40 sequentially acquired slices, slice thickness = 2 mm, interslice gap = 1 mm, TE = 25 ms, volume TR = 2 s, echo-spacing = 500 µs, BW = 2298 Hz/Px. The slice block was axial-to-coronal single oblique and aligned and centered manually with the calcarine fissure. For each subject, an axial, dual echo, gradient echo field map and a T1-weighted anatomical image (MPRAGE, resolution = (1 mm)^3^, TE = 3.72 ms, TR = 2000 ms, flip angle = 5°, TI = 1050 ms) were also acquired.

The k-space raw complex data from all time series were reconstructed using a trajectory-based reconstruction [19] followed by RSS calculation to combine the images from the 24 receiver channels. The resulting magnitude images were then processed using SPM8 (http://www.fil.ion.ucl.ac.uk/spm/, [22]) with additional routines implemented in Matlab. After discarding the first five volumes of each run, the EPI data were spatially co-registered to the first volume of the first run. The gradient echo field map was processed to create a voxel displacement map and used to correct the realigned images for geometric distortions [23]. Each subject’s anatomical image was registered to their corresponding undistorted, realigned EPI data and segmented into grey and white matter tissue probability maps using the unified segmentation procedure in SPM8 [24]. The resulting inverse spatial normalization parameters were used to match a brain atlas (AAL toolbox, [25]) defined in MNI space [26] to the anatomy of each subject and hence define a visual cortex (VC) ROI. Finally the ROIs were restricted to grey matter by masking with the grey matter tissue probability maps (from the segmentation step) after thresholding at a probability value greater than 0.01.

For each subject the noise measure (σ′_0_) was estimated from the thermal noise measurement (i.e. with no RF excitation). For the time series acquired with the different (non-zero) flip angles, maps of SNR′_0_ and tSNR were calculated as described previously for the phantom data. The mean SNR′_0_ and tSNR were calculated for each subject’s VC ROI and the mean and SD were estimated over subjects for each flip angle. The original noise model defined in equation 3 and the extended model defined in equation 6 were fitted to the mean tSNR and SNR′_0_ values. Values for λ and model fit sum-of-squares errors (SSE) were estimated for the two models and κ was estimated for the extended model only.

An independent estimate of actual SNR_0_ was also calculated for each subject and each flip angle time series using the complex trajectory-based reconstructed data from each channel in the first non-discarded image in the EPI time series and the noise covariance as described previously for the phantom data. The mean SNR_0_ was calculated for each subject’s VC ROI and the mean and SD were estimated over subjects for each flip angle. As for the SNR′_0_ values, the original and extended noise models were fitted to the mean tSNR and SNR_0_ values. Values for λ and model fit sum-of-squares errors (SSE) were estimated for the two models and κ was estimated for the extended model only.

## Results

### Accuracy and Precision of Model Fit Estimated from Monte Carlo Simulations

The Monte Carlo simulations of the accuracy and precision indicated that the non-linear model fit is an unbiased estimator, provided that the set of five SNR′_0_ values (over which the fitting is performed) are well distributed. Figure 1 shows the top 5% best sets of five SNR′_0_ values for achieving model fits with a minimal bias for different SNR′_0_ ranges (50–600 (a,b), 50–300 (c,d)) and κ values (1.4 (a,c) 1.8 (b,d)). Approximately speaking, unbiased fits were observed for SNR′_0_ sets which cover rather densely the low and high SNR′_0_ range and less densely the mid SNR′_0_ range. The bias in 1/λ and κ was always smaller than 1.2% when the best SNR′_0_ sets were chosen, indicating a high accuracy.

For the set of best SNR′_0_ values yielding minimal bias, the precision in estimating 1/λ was high with SD always smaller than 7.0 for the large SNR′_0_ range (50–600) and smaller than 11.3 for the small SNR′_0_ range (50–300). These SD values compared reasonably with the overall lowest SD of 2.7 and 4.5 achieved in all 5000 simulations of different SNR′_0_, i.e., the highest achievable precision. The precision in estimating κ was comparably lower, since the SD was always lower than 0.45 for the large SNR′_0_ range and 0.27 for the small SNR′_0_ range (compared to the minimal SD ranging between 0.12–0.15 for all simulations, i.e., the highest precision achievable).

For the gel phantom, Monte Carlo simulations demonstrated that it is possible to determine κ robustly with high precision and accuracy even when only relatively low values for SNR′_0_/κ were measured compared to 1/λ, i.e., a maximal SNR′_0_/κ = 180 compared to 1/λ = 1800. The maximal bias in κ was smaller than 2.3% and the SD was smaller than 0.085 for all simulated κ values from 1.0 to 2.0. Note that 1/λ values could not be reliably estimated as expected from the fact that only low SNR measurements informed the fit.

For a wide range of SNR_0_ values (SNR_0_ = 80–140) the regression analysis yielded a linear dependence (slope) of κ on 1/λ of 0.0025 (i.e., a change of 10 in 1/λ would result on average in a spurious change of 0.025 in κ). Similarly, the linear dependence of 1/λ on κ was 4.7 (i.e., a change of 0.5 in κ would result on average in a spurious change of 2.35 in 1/λ). Thus, the effect is rather small and confirms good estimability of the model under realistic conditions.

### Validation of Extended Model Fit using Phantom Data and Independent Estimate of Actual Image SNR

**Figure 2:**
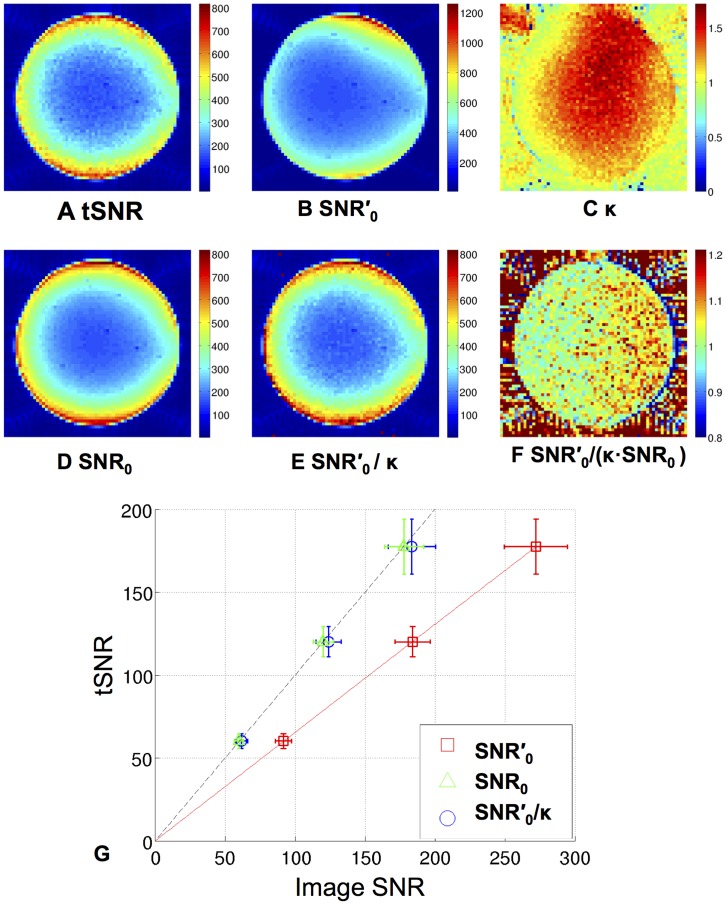
Validation of extended model fit for phantom data acquired at 3T using a 32-channel RF head receive coil. Results for a single central slice through phantom data show maps of A) tSNR, B) SNR′_0_ C) κ, D) SNR_0_ E) SNR′_0_/κ F) SNR′_0_/(κ ·SNR_0_)_._ The tSNR and SNR maps are shown for the EPI data acquired with the largest flip angle (90°). The κ map was estimated by fitting the extended model to each voxel of the tSNR and SNR′_0_ maps calculated from EPI time series acquired at 3 different flip angles (17°, 37° and 90°). In G, a plot of the mean and SD of tSNR against SNR′_0_ (red squares), SNR_0_ (green triangles) and SNR′_0_/κ (blue circles) within a 20×20 voxels ROI at the centre of the phantom is shown for all flip angle time series and the red solid line shows the fit of the extended model to the SNR′_0_ values. The black dashed line is the line of identity.

The results of the validation using phantom data and an independent estimate of actual image SNR are shown in Figure 2. Maps of tSNR, SNR′_0_, and SNR_0_ for the data acquired from the largest flip angle (90°) are shown in Figures 2a, b and d respectively and the map of κ values estimated from the fit of the extended model to the SNR′_0_ maps from all flip angles is shown in Figure 2c. In terms of magnitude and spatial distribution, the similarity between the tSNR and SNR_0_ maps and the difference between those and the SNR′_0_ map are apparent. However, voxelwise scaling of the SNR′_0_ map by the κ map (Figure 2c) resulted in a map (Figure 2e) that was comparable to both the tSNR and SNR_0_ maps. This result validates the proposed linear relationship between SNR′_0_ and SNR_0_ (equation 5). The map of the ratio SNR′_0_/(κ·SNR_0_) shown in Figure 2f confirmed that the κ value estimated from the phantom data satisfied the equation κ = SNR′_0_/SNR_0_ to within 4% averaged over the signal in the central slice of the phantom (mean ± SD of the ratio SNR′_0_/(κ·SNR_0_)_ = _1.04±0.1). The map of κ values ranged from a minimum of 0.4 up to 1.7 in the central slice of the phantom. Since SNR′_0_ is affected by noise correlations whereas SNR_0_ accounts for them, this result demonstrates the spatial variation of the impact of correlated noise. Figure 2g shows a plot of tSNR against SNR′_0_, SNR_0_ and SNR′_0_/κ (mean ± SD) within a 20×20 voxels ROI at the centre of the phantom for all flip angle time series. For this ROI, the fit of the extended model to the tSNR and SNR′_0_ values resulted in κ = 1.5±0.1 (mean ± SD) and the fit to the tSNR and SNR_0_ values resulted in κ = 1.0±0.04 (mean ± SD), since noise correlations were accounted for in SNR_0_. As expected from the results of the Monte Carlo simulation, the fit of the λ value was imprecise and biased with λ = 1/935.8±1/833.3 for the first fit and λ = 1/913.6±1/813.3 for the latter fit (mean ± SD).

### Fit of Extended Model to Human Task-free fMRI Data

**Figure 3:**
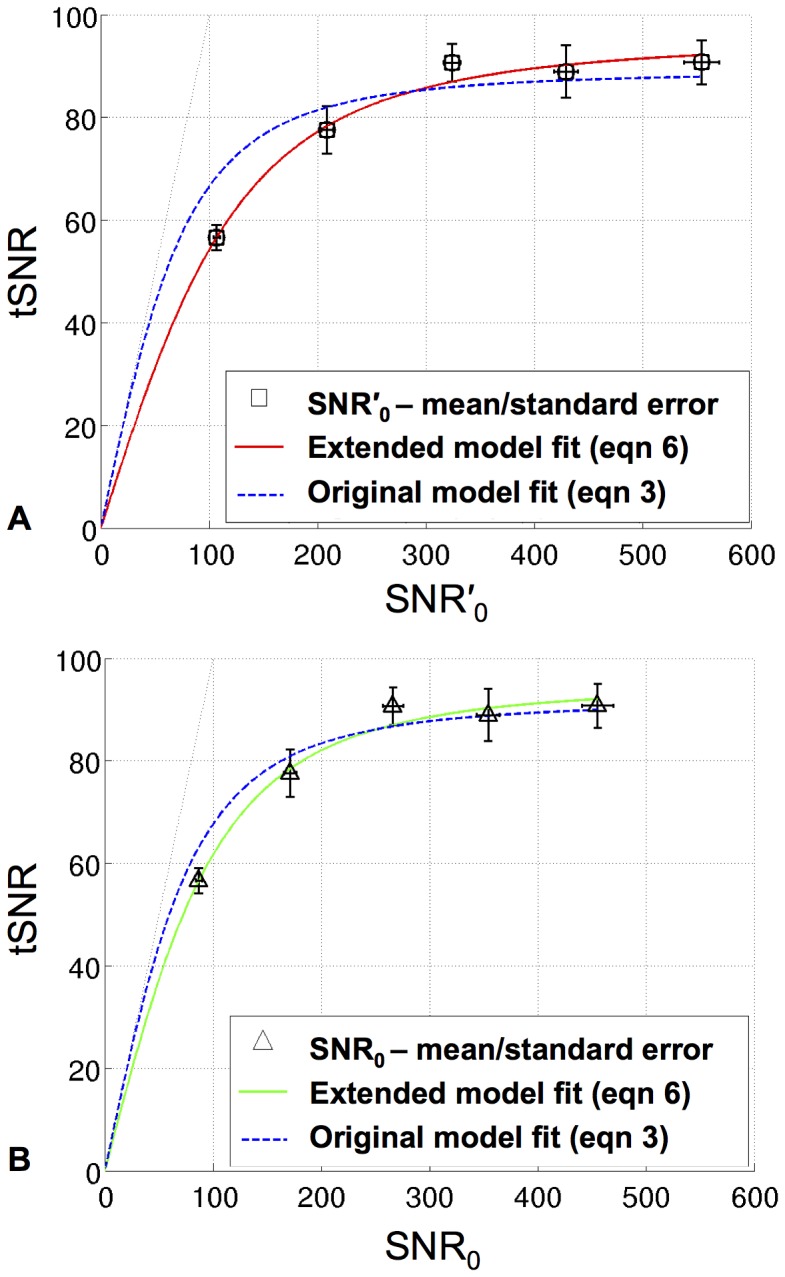
Fit of extended model to human task-free fMRI data acquired at 7T with a 24-channel receive head coil. A) Plot of tSNR against SNR′_0_ is shown for EPI time series acquired using 5 different flip angles (8°, 16°, 26°, 38° and 70°). Each data point (black squares) represents the mean and standard error over 5 subjects of mean tSNR and SNR′_0_ in the VC ROI. The solid red line represents the fit of the extended model (using equation 6) and the blue dashed line represents the fit of the original model (using equation 3). B) Plot of tSNR against the independently measured SNR_0_ for the same data as in (A). Black triangles represent the mean and standard error over 5 subjects, the solid green line represents the fit of the extended model (using equation 6) and the blue dashed line represents the fit of the original model (using equation 3).

**Figure 4:**
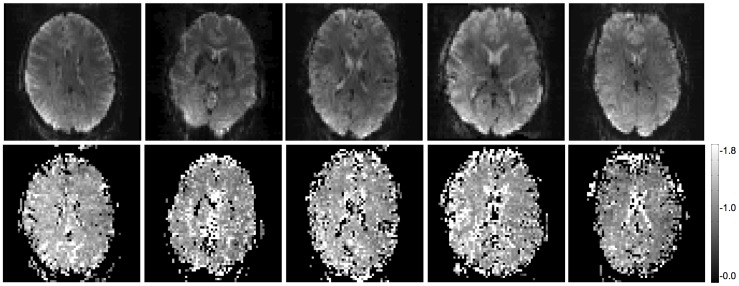
Subject specific maps of κ estimated from fit of extended model to human task-free fMRI data acquired at 7T with a 24-channel receive head coil (bottom row). The top row shows the corresponding subject specific EPI images acquired at the 70° flip angle.

Figure 3a shows plots of mean tSNR versus mean SNR′_0_ measured in the VC ROI of 5 subjects for the task-free fMRI data acquired at 7T. The parameters estimated from the fits of the two models are given in Table 1. The extended model using equation 6 (red solid curve) gave a much improved fit compared to the original model using equation 3 (blue dashed curve), with SSE = 17.8 and 191.5 respectively. This was particularly apparent at low to medium SNR′_0_ values and was reflected by the value estimated for the parameter κ = 1.5±0.2 (mean ± SD). Figure 4 shows the subject specific EPI images acquired at the 70° flip angle together with the maps of κ estimated for the fit of the extended model to the tSNR versus SNR′_0_ values at each voxel. The value estimated for 1/λ was slightly higher for the extended model compared to the original model (95.2±9.2 and 89.1±9.4 respectively, (mean ± SD)). Note that the difference between these values fell within the precision in estimating 1/λ for this SNR′_0_ range as predicted by the simulation results (i.e. SD <7.0).

**Table 1:** Parameters estimated from fit of extended model (equation 6) and original model (equation 3) to human task-free fMRI data acquired at 7T with a 24-channel receive head coil (mean ± standard deviation) and sum-of-squares errors (SSE) indicating goodness-of-fit of the models.

	*Extended model (* *equation 6* *)*			*Original model (* *equation 3* *)*	
	1/λ	κ	SSE	1/λ	SSE
*tSNR vs SNR′_0_*	95.2±9.2	1.5±0.2	17.8	89.1±9.4	191.5
*tSNR vs SNR_0_*	95.1±9.1	1.2±0.1	17.6	91.7±9.5	68.6

Parameters are given for model fits to tSNR versus SNR′_0_ (data shown in Figure 3a) and tSNR versus SNR_0_ (data shown in Figure 3b).

Figure 3b shows plots of tSNR against the actual image SNR (SNR_0_) for the same data as in Figure 3a. The fits of the extended model (solid green line) and the original model (blue dashed line) are shown for these data and the parameters are given in Table 1. For these results, the fit of the extended model was still improved compared to the original model (SSE = 17.6 and 68.8 respectively), even though one may have expected the original model to describe the data as well as the extended model since actual image SNR values were used. The plots show that again the fit of the original model was more problematic at low to medium SNR_0_ values. This was also highlighted by the fact that the κ value estimated from the extended model fit was not 1.0 as expected, but 1.2±0.1 (mean ± SD). However, it should also be noted that the difference in estimates of 1/λ for the two models was not greater than their SD values.

## Discussion

The model introduced by Krueger and Glover [1] describes the tSNR of an EPI time series as a function of image SNR. It can be used to estimate the maximum attainable tSNR (1/λ) or BOLD sensitivity for a given set of MR acquisition parameters [2] and image processing strategies [4]. This model requires that the image SNR be estimated accurately, which is not straightforward for data acquired using multi-channel coils. First of all, access to the k-space raw data is necessary which can be cumbersome and often practically impossible for large numbers of receiver coils (e.g. up to 30 GB per EPI time series in this study). Secondly, the steps required to correctly scale the image SNR measurements are complicated, and may require corrections for the RSS combination and magnitude operation, as well as for noise correlations between receiver coils and the algebraic reconstruction. The complexity and practical challenge are reflected by extensive discussion in the literature [3], [5], [6], [8], [10], [11], [17]. In this work we have proposed an extension to this model that allows the tSNR of an EPI time series acquired using multi-channel receiver coils to be characterized by estimating an apparent image SNR value (SNR′_0_) simply and directly from non-accelerated RSS combined images. The extended model includes an additional parameter κ, which scales the apparent image SNR to be equal to the actual SNR (SNR_0_). Since the apparent SNR′_0_ does not account for noise correlations between receiver coils whereas the actual SNR_0_ accounts for them, the parameter κ reflects the impact of correlation on the data.

### Robustness of the Extended Model Studied with Monte Carlo Simulations

The Monte Carlo simulations showed that the extended model parameters λ and κ can be accurately determined without significant bias from a series of five SNR′_0_ and tSNR measurements when the set of SNR′_0_ values is appropriately chosen. In other words, tSNR should be measured at high and low SNR′_0_ values with less emphasis on the medium SNR′_0_ range (Figure 1). Based on only 5 measurements, a reasonable precision for the estimated 1/λ is achieved with a coefficient of variation of ∼5–10%. However, the precision of κ estimates is somewhat low with coefficients of variation between 20–30%. This is less critical when the extended model is used for studies on the impact of physiological noise whose primary outcome measure is 1/λ. A higher precision in κ could be achieved by increasing the sampling of SNR′_0_ values. Furthermore, a robust estimation of κ (coefficient of variation ∼5%) can be achieved even when the maximal SNR′_0_ is low compared to 1/λ (i.e. when λ approaches 0 as it can for phantom data when the MRI scanner is stable). In this case, estimates of 1/λ are not reliable. For typical SNR_0_ values observed in vivo at 7T, the interdependence between estimates of 1/λ and κ was low, corroborating a good estimability of the model.

We would like to note that the original model proposed by Krueger and Glover [1] shows similar characteristics in estimating 1/λ and also relies on a sufficient range of SNR′_0_ values to avoid bias in its estimation.

### Validation of Extended Model using Phantom Data

The fit of the extended model to the phantom data resulted in a map of κ values that accurately (within 4%) scaled the map of apparent SNR values (SNR′_0_) to be equal to an independent measure of the actual image SNR (SNR_0_) calculated using an established method [3], [5], [6]. The results showed that κ estimated for SNR′_0_ was spatially highly variable (>50% change) making the extended model essential for bias free estimates. The spatial variability of κ can be well explained by the spatial variation of noise in composite images as a result of noise correlations between the different channels of the receiver system [13], [14]. When the extended model was fitted to the estimate of actual image SNR, the estimate for κ was as expected 1.0±0.01 (mean ± SD). Note that noise correlations between the different channels of the receiver system were taken into account for the estimate of SNR_0_ via the noise covariance between all pairs of receiver channels (i.e. equation 8). The large variability of values estimated for 1/λ for the two data sets could be explained by the results of the Monte Carlo simulations, which demonstrated that 1/λ values could not be reliably estimated when all measured SNR values are low compared to 1/λ.

### Fit of Extended Model to Human Task-free fMRI Data

The results for the human data showed a much improved fit of the extended model to tSNR and SNR′_0_ measured from non-accelerated RSS combined images, compared to the original model by Krueger and Glover [1]. Although the estimate of 1/λ was very similar for the two models (within the SD values), the fit of the extended model was particularly improved for low to medium values of SNR′_0._ This suggests that at low to medium SNR′_0_ values, the original model may lead to incorrect estimates of achievable tSNR if SNR values are not measured accurately. This is particularly important for optimizing EPI parameters for this lower SNR range where thermal noise components contribute significantly, e.g. by varying spatial resolution [2], [27] or flip angle [28]. Using the extended model to characterize the tSNR as a function of apparent image SNR (SNR′_0_) estimated from RSS combined images avoids these potential problems.

Notably, when actual SNR values (SNR_0_) were calculated using the established method, the extended model still resulted in a better fit than the original model and the estimate for κ was slightly higher than expected (1.2±0.1, mean ± SD versus 1.0). Possible reasons for this discrepancy are discussed below under methodological considerations. Interestingly, the variation in estimates of 1/λ for both SNR measures and both models was within the given precision. This highlights the relatively high accuracy of estimates of 1/λ for both the original and extended models, suggesting that in general these models are rather robust for estimating the maximum attainable tSNR for a given set of MR acquisition parameters.

### Methodological Considerations

The validation of the extended model using phantom data clearly demonstrated that the estimate of κ satisfied the equation κ = SNR′_0_/SNR_0_ when SNR_0_ was calculated using an established independent measure. This was less clear-cut for the human data. A discrepancy of between 10% and 30% was observed between SNR′_0_/κ and the independently measured SNR_0_, possibly due to the limited precision of the κ estimates. The discrepancy might be reduced by sampling more SNR′_0_ values. Furthermore, κ was estimated for tSNR and SNR′_0_ values averaged over an ROI for each subject, which could further decrease its precision due to the presence of outliers or effects of non-linearity (i.e. averaging before the non-linear fit). Examples of these are apparent in the estimated maps of κ shown in Figure 4.

The discrepancy may also have been related to limitations in both the original and the extended noise models. For example, the original model assumes that tSNR ≈ SNR_0_ at lower SNR_0_ values, (i.e. where noise is dominated by thermal noise). However, the estimation of tSNR in human data requires motion correction, which may alter the relationship between tSNR and SNR_0_. Furthermore, correlations between the physiological noise from different channels may have an effect on the tSNR, e.g. by spatial resampling, which is not included in the original model presented here and is also not accounted for by the image reconstruction. These two latter effects may influence the estimate of κ from human EPI time series data in the extended model. As shown in Figure 4, estimates for κ are much higher around the edges of the brain, and in the ventricles and other fluid spaces, where pulsatile motion from fluid and head movement are typically most apparent.

In this work, the parameter κ has been interpreted as a factor which accounts for the effect of noise correlations by scaling the apparent image SNR (SNR′_0_) so that it equals the actual SNR (SNR_0_). In theory, κ could be predicted from the receiver coils’ flux lines and noise correlation coefficients. Practically this is complicated by large numbers of receiver coils, their geometric arrangement, electrical coupling properties and differing sensitivity profiles [8], [13], [14].

Many methods have been proposed to estimate the noise and hence the SNR in MR images (for example see [5] for a detailed review of methods). The noise measurement method used in this work (i.e. using equation 4) was selected because it can be estimated from a short thermal noise reference scan and has been shown to be independent of noise correlations [8]. Furthermore, it does not require any pulse sequence or image reconstruction modifications. It should be pointed out that this noise measure and the proposed extended model is applicable to all situations where there is a constant scale factor between the measured apparent SNR′_0_ and the actual SNR_0_ [5], although the spatial variation of κ is likely to be different for different situations. For example, the extended model could be used to characterize the tSNR and physiological noise properties of GRAPPA parallel imaging acquisitions [7] and reconstructions because g-factors and under-sampling factors introduce a constant scaling factor between apparent and actual SNR. The extended model in its current form is not valid for low SNR values (<50), if 32 or more channels are used since correction factors must be applied to account for changes in the noise distribution [8]. However, in principle a correction for low SNR values could be added to the model [3], [8], although an iterative estimation approach may be necessary.

### Conclusion

We have presented a simple extension to the noise model of [1] which allows the characterization of tSNR and SNR of non-accelerated RSS combined images from multi-channel receiver coils without needing to handle raw data or to perform arduous custom-made image reconstruction. To our knowledge, no other published methods can estimate the model proposed in [1] from RSS combined magnitude data from multi-channel receiver coils. We derive the proposed extended model from basic physical principles and show that our simple SNR correction is valid for non-accelerated data with an SNR >50 and for multi-channel receiver coils with ≤32 channels. Given these constraints, the model can be applied to all situations where a constant scaling factor exists between the actual SNR_0_ and the apparent SNR′_0_ measured in the reconstructed and combined images (e.g. non-accelerated, RSS combined images). We successfully validated the model by Monte Carlo simulations as well as application to phantom data at 3T and task-free human fMRI data at 7T. In particular, the extended model improves the prediction of low to medium tSNR values, which is especially important for optimizing high-resolution fMRI experiments, where thermal noise components contribute significantly. In summary, the extended model offers a simple and robust method to investigate the fundamentals of physiological noise, to assess the impact of physiological noise correction and image reconstruction and to optimize EPI acquisition parameters.

## References

[1] Krueger G, Glover GH (2001) Physiological noise in oxygenation-sensitive magnetic resonance imaging. Magn Reson Med 46 (4): 631–637. 10.1002/mrm.1240 11590638

[2] Triantafyllou C, Hoge RD, Krueger G, Wiggins CJ, Potthast A, et al (2005) Comparison of physiological noise at 1.5 T, 3 T and 7 T and optimization of fMRI acquisition parameters. Neuroimage 26 (1): 243–250. 10.1016/j.neuroimage.2005.01.007 15862224

[3] Triantafyllou C, Polimeni JR, Wald LL (2011) Physiological noise and signal-to-noise ratio in fMRI with multi-channel array coils. Neuroimage 55 (2): 597–606. 10.1016/j.neuroimage.2010.11.084 PMC3039683 21167946

[4] Hutton C, Josephs O, Stadler J, Featherstone E, Reid A, et al (2011) The impact of physiological noise correction on fMRI at 7 T. Neuroimage 57. (1): 101–112. 10.1016/j.neuroimage.2011.04.018 PMC3115139 21515386

[5] Kellman P, McVeigh ER (2005) Image reconstruction in SNR units: a general method for SNR measurement. Magn Reson Med 54 (6): 1439–1447. 10.1002/mrm.20713 PMC2570032 16261576

[6] Roemer PB, Edelstein WA, Hayes CE, Souza SP, Mueller OM (1990) The NMR phased array. Magn Reson Med 16 (2): 192–225. 10.1002/mrm.1910160203 2266841

[7] Griswold MA, Jakob PM, Heidemann RM, Nittka M, Jellus V, et al (2002) Generalized autocalibrating partially parallel acquisitions (GRAPPA). Magn Reson Med 47 (6): 1202–1210. 10.1002/mrm.10171 12111967

[8] Constantinides CD, Atalar E, McVeigh ER (1997) Signal-to-noise measurements in magnitude images from NMR phased arrays. Magn Reson Med 38 (5): 852–857. 10.1002/mrm.1910380524 PMC2570034 9358462

[9] Wiggins GC, Triantafyllou C, Potthast A, Reykowski A, Nittka M, et al (2006) 32-channel 3 Tesla receive-only phased-array head coil with soccer-ball element geometry. Magn Reson Med 56 (1): 216–223. 10.1002/mrm.20925 16767762

[10] Dietrich O, Raya JG, Reeder SB, Reiser MF, Schoenberg SO (2007) Measurement of signal-to-noise ratios in MR images: influence of multichannel coils, parallel imaging, and reconstruction filters. J Magn Reson Imaging 26 (2): 375–385. 10.1002/jmri.20969 17622966

[11] Gilbert G (2007) Measurement of signal-to-noise ratios in sum-of-squares MR images. J Magn Reson Imaging 26 (6): 1678. 10.1002/jmri.21171 18059007

[12] Koay CG, Basser PJ (2006) Analytically exact correction scheme for signal extraction from noisy magnitude MR signals. J Magn Reson 179 (2): 317–322. 10.1016/j.jmr.2006.01.016 16488635

[13] Redpath TW (1992) Noise correlation in multicoil receiver systems. Magn Reson Med 24 (1): 85–89. 10.1002/mrm.1910240109 1556932

[14] Hayes CE, Roemer PB (1990) Noise correlations in data simultaneously acquired from multiple surface coil arrays. Magn Reson Med 16 (2): 181–191. 10.1002/mrm.1910160202 2266840

[15] Edelstein WA, Glover GH, Hardy CJ, Redington RW (1986) The intrinsic signal-to-noise ratio in NMR imaging. Magn Reson Med 3 (4): 604–618. 10.1002/mrm.1910030413 3747821

[16] Friedman L, Glover GH (2006) Report on a multicenter fMRI quality assurance protocol. J Magn Reson Imaging 23 (6): 827–839. 10.1002/jmri.20583 16649196

[17] Greve DN, Mueller BA, Liu T, Turner JA, Voyvodic J, et al (2011) A novel method for quantifying scanner instability in fMRI. Magn Reson Med 65 (4): 1053–1061. 10.1002/mrm.22691 PMC3117086 21413069

[18] Weiskopf N, Hutton C, Josephs O, Deichmann R (2006) Optimal EPI parameters for reduction of susceptibility-induced BOLD sensitivity losses: a whole-brain analysis at 3 T and 1.5 T. Neuroimage 33. (2): 493–504. 10.1016/j.neuroimage.2006.07.029 16959495

[19] Josephs O, Deichmann R, Turner R (200) Trajectory measurement and generalised reconstruction in rectilinear EPI. In: Proceedings of ISMRM 8, Denver, Colorado, USA.

[20] Wright PJ, Mougin OE, Totman JJ, Peters AM, Brookes MJ, et al (2008) Water proton T1 measurements in brain tissue at 7, 3, and 1.5 T using IR-EPI, IR-TSE, and MPRAGE: results and optimization. MAGMA 21 (1–2): 121–130. 10.1007/s10334-008-0104-8 18259791

[21] Lutti A, Hutton C, Stadler J, Josephs O, Speck O et al.. (2010) Radio frequency (B1) field mapping at 7T using 3D SE/STE EPI technique. In: Proceedings of Joint Annual Meeting ISMRM-ESMRM, Stockholm, Sweden.

[22] Friston K. Statistical Parametric Mapping: The Analysis of Functional Brain Images. (2007) In:Friston K, Ashburner J, Kiebel S, Nichols T, Penny W, editors. Statistical Parametric Mapping. London: Elsevier Ltd. 10–31.

[23] Hutton C, Bork A, Josephs O, Deichmann R, Ashburner J, et al (2002) Image distortion correction in fMRI: A quantitative evaluation. Neuroimage 16 (1): 217–240. 10.1006/nimg.2001.1054 11969330

[24] Ashburner J, Friston KJ (2005) Unified segmentation. NeuroImage 26 (3): 839–851. 10.1016/j.neuroimage.2005.02.018 15955494

[25] Tzourio-Mazoyer N, Landeau B, Papathanassiou D, Crivello F, Etard O, et al (2002) Automated anatomical labeling of activations in SPM using a macroscopic anatomical parcellation of the MNI MRI single-subject brain. Neuroimage 15 (1): 273–289. 10.1006/nimg.2001.0978 11771995

[26] Evans A, Collins DL, Mills SR, Brown ED, Kelly RL, et al.. (1993) 3D statistical neuroanatomical models from 305 MRI volumes [abstract]. In: Anonymous. 1813–1817.

[27] Bodurka J, Ye F, Petridou N, Murphy K, Bandettini PA (2007) Mapping the MRI voxel volume in which thermal noise matches physiological noise–implications for fMRI. Neuroimage 34 (2): 542–549. 10.1016/j.neuroimage.2006.09.039 PMC1815476 17101280

[28] Gonzalez-Castillo J, Roopchansingh V, Bandettini PA, Bodurka J (2011) Physiological noise effects on the flip angle selection in BOLD fMRI. Neuroimage 54 (4): 2764–2778. 10.1016/j.neuroimage.2010.11.020 PMC3020268 21073963

