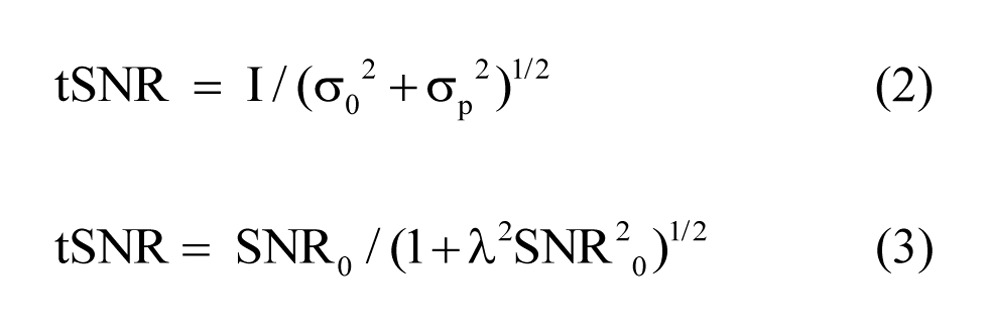# Correction: Modelling Temporal Stability of EPI Time Series Using Magnitude Images Acquired with Multi-Channel Receiver Coils

**DOI:** 10.1371/annotation/ad7981aa-87ee-458a-ae72-00972b3af933

**Published:** 2013-03-13

**Authors:** Chloe Hutton, Evelyne Balteau, Antoine Lutti, Oliver Josephs, Nikolaus Weiskopf

There were errors in Equations 2 and 3. The 2 letters "s" in Equation 2 should both be "σ" symbols. The letter "l" in Equation 3 should be a "λ". Please view the complete, correct equation here: